# A case of colorectal large cell neuroendocrine carcinoma accompanied by disseminated peritoneal leiomyomatosis

**DOI:** 10.1186/s40792-020-01069-4

**Published:** 2020-12-09

**Authors:** Kunihiko Suga, Hiroomi Ogawa, Makoto Sohda, Chika Katayama, Naoya Ozawa, Katsuya Osone, Takuhisa Okada, Takuya Shiraishi, Ryuji Katoh, Akihiko Sano, Makoto Sakai, Takehiko Yokobori, Ken Shirabe, Hiroshi Saeki

**Affiliations:** 1 Department of General Surgical Science, Graduate School of Medicine, Gunma University, 3-39-22 Showa-machi, Maebashi-shi, Gunma-ken 371-8511 Japan; 2 Division of Integrated Oncology Research, Gunma University Initiative for Advanced Research (GIAR), 3-39-22 Showa-machi, Maebashi, Gunma Japan

**Keywords:** Large cell neuroendocrine carcinoma, Neuroendocrine tumor of the colon, LCNEC

## Abstract

**Background:**

Neuroendocrine carcinomas (NECs) of the colon are among the rarest types of colorectal cancers. Among these, large cell type neuroendocrine carcinoma (LCNEC) is particularly rare. Colorectal NEC is an aggressive disease, and there are few reports of long-term survivors. Here, we report a case of LCNEC accompanied by disseminated peritoneal leiomyomatosis that was difficult to diagnose.

**Case presentation:**

The case involves a 62-year-old female found to be positive for fecal occult blood by medical examination. An endoscopy revealed a tumor in the ascending colon, and the biopsy revealed poorly differentiated cancer. Abnormal FDG accumulation with peritoneal thickening was visible on 18F-fluorodeoxyglucose positron-emission tomography (FDG-PET) and suspected to be peritoneal dissemination. Laparoscopic ileocecal resection was performed for the tumor of the ascending colon with abdominal wall invasion. At that time, numerous intra-abdominal nodules were observed, indicating peritoneal dissemination. The pathological diagnosis of the primary lesion was LCNEC, and the patient requested to undergo total peritoneal resection. After one course of chemotherapy with irinotecan plus cisplatin, she underwent total peritoneal resection, uterine annex resection, left inguinal lymph node resection, and intra-abdominal hyperthermic intraperitoneal chemotherapy with mitomycin C. Because a postoperative pathological examination revealed that the intra-abdominal nodules were leiomyomas, we diagnosed the patient with disseminated peritoneal leiomyomatosis. The left inguinal lymph node was diagnosed with a metastatic tumor. In summary, the final diagnosis was LCNEC in the ascending colon with inguinal lymph node metastasis. Postoperative chemotherapy has been administered to date. She is currently 18 months post-primary surgery and 15 months post-peritonectomy without apparent recurrence or metastatic findings.

**Conclusion:**

We experienced a case of Stage IVa colorectal LCNEC accompanied by disseminated peritoneal leiomyomatosis. Although the prognosis is generally poor, multidisciplinary treatment for advanced colorectal LCNEC may result in a favorable outcome for some patients. If peritoneal dissemination is suspected during operation, sampling of the nodule to confirm the pathological diagnosis is advisable.

## Background

Neuroendocrine carcinomas (NECs) of the colon are rare, accounting for about 0.03% of all colorectal cancers [1]. Among these, large cell type neuroendocrine carcinoma (LCNEC) is particularly rare. The prognosis for colorectal NEC is poor, with an overall survival of 10.5 months and a 1-year survival rate of 46% [1].

Multiple benign nodules in the abdominal cavity can be misdiagnosed as malignant peritoneal dissemination when they are accompanied by cancers. Disseminated peritoneal leiomyomatosis (DPL) is characterized by the disseminated intraperitoneal development of leiomyoma tissue and can be a differential disease of peritoneal dissemination [2].

In this report, we describe a case of LCNEC accompanied by DPL that was clinically difficult to diagnose.

## Case presentation

**Fig. 1:**
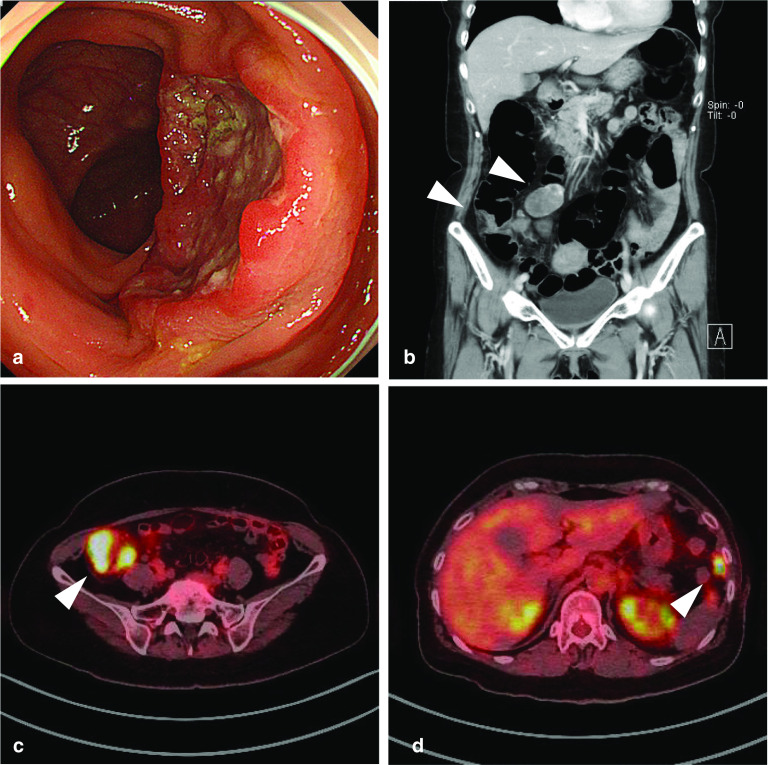
**a** Colonoscopy showed a semicircular type 3 tumor in the ascending colon. **b** CT showed a mass in the ascending colon with regional lymph node enlargement (arrowheads). **c**, **d** FDG-PET showed abnormal FDG uptake in the lesion in the ascending colon and peritoneal thickening of the left upper abdomen

**Fig. 2:**
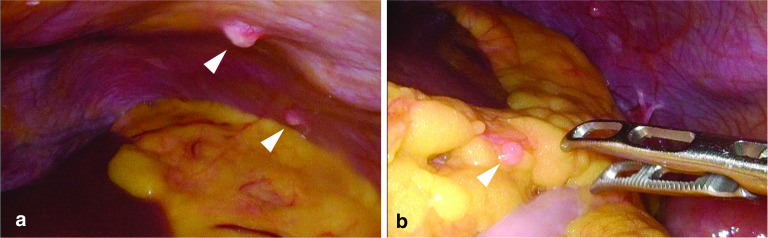
Intra-abdominal nodules recognized during laparoscopic surgery in the **a** left upper abdomen and **b** greater omentum (arrowheads)

The case involves a 62-year-old woman found to be positive for fecal occult blood by medical examination. An endoscopy by her previous doctor showed a tumor in the ascending colon, and the biopsy showed poorly differentiated cancer. The patient was referred to our institution for surgery. Her blood test results were as follows: carcinoembryonic antigen (CEA), 76.1 ng/mL (normal range < 5.0 ng/mL) and carbohydrate antigen (CA) 19-9, 192 U/mL (normal range < 37 U/mL). A colonoscopy showed a semicircular type 3 tumor contralateral to the Bauhin valve in the ascending colon (Fig. 1a). A biopsy supported a diagnosis of poorly differentiated adenocarcinoma or NEC. Abdominal computed tomography (CT) showed wall thickening with contrast effect in the ascending colon, enlargement of the peripheral lymph nodes (Fig. 1b) and a uterine leiomyoma. On an 18F-fluorodeoxyglucose positron-emission tomography (FDG-PET) scan, we observed FDG uptake at the lesion in the wall of the ascending colon (standardized uptake value[SUV]_max_ 22.9), peripheral lymph nodes (SUV_max_ 23.8), peritoneum of the left upper abdomen (SUV_max_ 13.4), and pelvic floor on the left dorsal side (SUV_max_ 5.2) (Fig. 1c, d). From these results, the tumor was preoperatively diagnosed as an ascending colon malignant neoplasm with peritoneal dissemination. The ileocecal resection of the specimen was performed to confirm the diagnosis. At the time of the operation, numerous intra-abdominal nodules were observed, indicating peritoneal dissemination (Fig. 2a, b), although a pathological diagnosis was not performed.

**Fig. 3:**
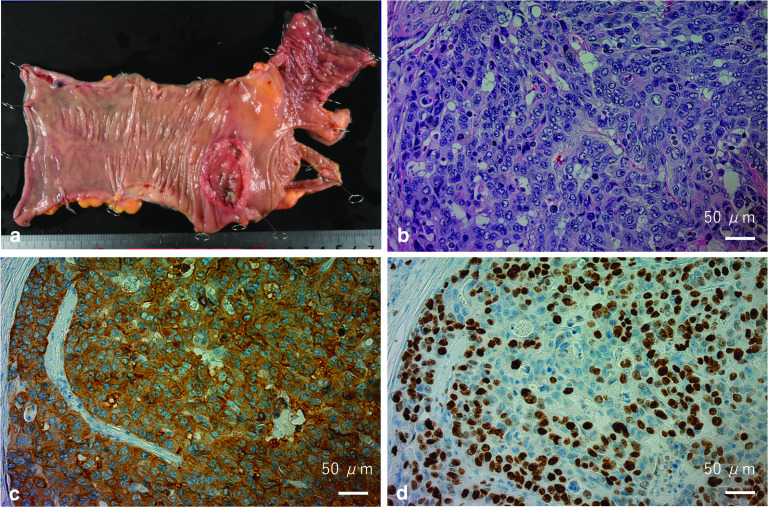
**a** Resected specimen of the semicircular type 3 tumor in the ascending colon. Histological findings with **b** hematoxylin and eosin staining, **c** synaptophysin, and **d** MIB-1 immunohistochemical staining

Postoperative pathological findings showed no adenoma or adenocarcinoma component in the tumor of the ascending colon. Immunohistochemical analysis was performed, and the results were as follows: keratin-positive, chromogranin-negative, synaptophysin-positive, CD56-positive, alcian blue/periodic acid Schiff-negative, and MIB-1 index of 57% (Fig. 3b–d). Based on these results, we ultimately diagnosed the patient with LCNEC. She had a good postoperative course and was discharged from the hospital 10 days after surgery.

Because of the diagnosis of peritoneal dissemination, the patient requested to undergo a total peritoneal resection at another hospital. After one course of chemotherapy (irinotecan plus cisplatin), FDG-PET re-examination showed FDG uptake in the left inguinal lymph node. She underwent a total peritoneal resection, uterine adnexal resection, removal of the left inguinal lymph node, and hyperthermic intraperitoneal chemotherapy (mitomycin C). Postoperative pathological examination revealed that the peritoneal nodules diagnosed as leiomyoma without any malignant findings, with spindle-shaped cell intermingling with eosinophilic spore bodies. The left inguinal lymph node was diagnosed with a metastatic tumor from LCNEC. In summary, the final diagnosis was LCNEC in the ascending colon with inguinal lymph node metastasis accompanied by DPL. Following two courses of irinotecan plus cisplatin as postoperative chemotherapy, she has received capecitabine monotherapy since March 2019. As of 18 months post-primary surgery and 15 months post-peritonectomy she was free of apparent recurrence or metastatic findings.

## Discussion

The definition and classification of NEC have changed over time, and according to the latest 2019 WHO classification, NEC is defined as a Ki-67 labeling index > 20% or cell division rate > 20 cells per 10 high power fields and categorized into the small and large cell types [3]. In the large intestine, the incidence of NEC is very low, accounting for ~ 0.03% of all colorectal cancers [1], and LCNEC is extremely rare. Mixed neuroendocrine–non-neuroendocrine neoplasms (MiNENs) are defined in the WHO classification as lesions with a neuroendocrine component (neuroendocrine tumor and NEC) and non-neuroendocrine component (adenocarcinoma, acinar cell carcinoma, squamous cell carcinoma, and others) comprising at least 30% of the total tumor mass. The case in this report was diagnosed with pure LCNEC without an adenocarcinoma component.

There are few previous reports on colorectal LCNECs, excluding MiNENs, with only 12 cases reported in English literature (Table 1) [4–12]. Most patients with Stage IV died within a few months after diagnosis. Only one patient with localized peritoneal dissemination survived beyond 3 years [11]. The present case is the only report of extra-regional lymph node metastasis. Even though this is a case with inguinal lymph node metastasis, the patient survives for almost 2 years. To the best of our knowledge, this patient in this case is the longest-surviving patient following the resection of metastatic inguinal lymph nodes. For cases involving oligometastases from LCNEC, surgical resection may be a possible option.

**Table 1:** Clinicopathologic findings of LCNEC of the colorectum that previously reported

Year	Author	Age	Gender	Biopsy	Location	LN metastasis	Distant metastasis	Depth of invasion	Stage	Ki-67	Outcome
2005	Kato	69	Female	–	A	n/a	Liver	SI (pancreas, duodenum)	IV	> 50%	7 M death
2010	Park	72	Female	LCNEC	A	7/20		SE	III	n/a	n/a
2011	Pascarella	74	Female	–	A	17/24		SE	III	90%	2 W death
2011	Kim	70	Male	–	A	11/29		SE	III	n/a	n/a
2012	Jukić	74	Male	n/a	C	11/14		SS	IV	50%	n/a
		94	Male	n/a	A	1/15		MP	III	80%	n/a
		58	Female	n/a	D	4/7		SS	IV	60%	n/a
		63	Male	n/a	R	5/5		SS	III	50%	n/a
		75	Female	n/a	R	2/18		SS	III	40%	n/a
		44	Female	n/a	A	0/10		SS	II	80%	n/a
		67	Female	n/a	R	1/11		SS	III	60%	n/a
		80	Female	n/a	C	8/8		SS	III	50%	n/a
2014	Minocha	63	Male	LCNEC	R	n/a	Liver, lung	n/a	IV	50%	1 M death
2014	Xu	66	Male	–	T	0/1	Liver	SS	IV	> 95%	2 M death
2018	Kim	74	Male	Poorly	R	13/20	Localized peritoneum	SE	IV	90%	3Y alive
2019	Chetty	79	Female	n/a	A	4/22		SS	III	95%	4Y death
		85	Female	n/a	A	3/25		SS	III	100%	5Y death
		89	Female	n/a	R	3/20		SS	III	80%	1Y alive

*A* ascending colon, *C* cecum, *D* descending colon, *LCNEC* large cell type neuroendocrine carcinoma, *LN* lymph node, *M* month, *MP* muscularis propria, *n/a* not available, *poorly* poorly differentiated adenocarcinoma, *R* rectum, *S* Sigmoid colon, *SE* serosa, *SI* tumor invades adjacent structures, *SS* subserosa, *T* transverse colon, *W* week, *Y* year

A definitive diagnosis of NEC was not made based on the previous doctor’s biopsy. In previous reports, NEC cases were difficult to diagnose by biopsy [11, 13]. Although resection of the primary tumor is not recommended for asymptomatic Stage IV colorectal cancer in Japanese Society for Cancer of the Colon and Rectum guidelines for the treatment of colorectal cancer [14], if the diagnosis based on biopsy is difficult to confirm, total resection of the primary tumor for both the diagnosis and treatment should be considered.

Preoperatively, FDG accumulation was observed in the left upper abdomen and in the pelvic floor. Taken together with the intraoperative findings, we judged that the patient had peritoneal dissemination. However, the postoperative pathological diagnosis revealed no peritoneal dissemination of NEC. The pathological diagnosis was leiomyoma, and then we finally diagnosed our patient with DPL [15]. Etiology of DPL is still unclear, but hormonal, genetic and metaplasia main theories proposed [15]. Additionally, iatrogenic factors after morcellation of myoma during laparoscopic surgery were reported as the main theory [16]. The cause of dissemination was unknown because there was no history of surgery, although uterine fibroids were present in this case. In addition, DPL is relatively rare and difficult to differentiate from peritoneal dissemination when it is complicated by neoplasia [17]. Based on the diagnosis with PET–CT and the association of the primary tumor with abdominal wall invasion, we strongly suspected the existence of peritoneal dissemination of LCNEC. A sampling of the nodule at the time of the initial surgery should have been considered for this case. Histological diagnosis of the nodule is necessary for the accurate differential diagnosis of intraperitoneal nodules. Although unlikely, it is possible that the disseminated nodules of NEC may have disappeared because the chemotherapy was administered prior to the total peritoneal resection.

With regard to the use of chemotherapy for colorectal NEC, there are reports of the successful use of etoposide plus cisplatin [18–21] and irinotecan plus cisplatin [20] therapy, depending on the regimen being used to treat colorectal cancer [4]. Although the evidence for chemotherapy is scarce, there are reports of surgery and chemotherapy contributing to better prognosis [22]. In the current case, the patient has continued taking an oral regimen (capecitabine) following irinotecan plus cisplatin therapy. Although no recurrence has been noted to date, further careful follow-up is needed.

## Conclusion

We experienced a case of colorectal LCNEC accompanied by disseminated peritoneal leiomyomatosis that was difficult to diagnose. Tumors diagnosed as poorly differentiated carcinomas by biopsy should be treated considering NEC as a differential diagnosis. Multidisciplinary treatment for advanced colorectal LCNEC may result in favorable outcomes for some patients with oligometastases. Peritoneal disseminated nodules should be investigated thoroughly.

## Data Availability

All data generated or analyzed during this study are included in this published article.

## Abbreviations

CA19-9: Carbohydrate antigen 19-9
CEA: Carcinoembryonic antigen
CT: Computed tomography
FDG-PET: Fluorodeoxyglucose-positron emission tomography
DPL: Disseminated peritoneal leiomyomatosis
HPF: High-power field
LCNEC: Large cell type neuroendocrine carcinoma
MiNEN: Mixed neuroendocrine–non-neuroendocrine neoplasms
NEC: Neuroendocrine carcinoma
NET: Neuroendocrine tumor
PAS: Periodic acid–Schiff
SUV: Standardized uptake value

## References

[1] Bernick PE, Klimstra DS, Shia J, Minsky B, Saltz L, Shi W (2004). Neuroendocrine carcinomas of the colon and rectum. Dis Colon Rectum.

[2] Demir M (2007). Disseminated peritoneal leiomyomatosis: magnetic resonance imaging and differential diagnosis. Australas Radiol.

[3] Klimstra D, Klöppel G, Larosasalas B (2019). Classification of neuroendocrine neoplasms of the digestive system. WHO Classification of Tumours Digestive System Tumours.

[4] Kato T, Terashima T, Tomida S, Yamaguchi T, Kawamura H, Kimura N (2005). Cytokeratin 20-positive large cell neuroendocrine carcinoma of the colon. Pathol Int.

[5] Park JS, Kim L, Kim CH, Bang BW, Lee DH, Jeong S (2010). Synchronous large-cell neuroendocrine carcinoma and adenocarcinoma of the colon. Gut Liver.

[6] Kim YN, Park HS, Jang KY, Moon WS, Lee DG, Lee H (2011). Concurrent large cell neuroendocrine carcinoma and adenocarcinoma of the ascending colon: a case report. J Korean Soc Coloproctol.

[7] Pascarella MR, McCloskey D, Jenab-Wolcott J, Vala M, Rovito M, McHugh J (2011). Large cell neuroendocrine carcinoma of the colon: a rare and aggressive tumor. J Gastrointest Oncol.

[8] Jukić Z, Limani R, Luci LG, Nikić V, Mijić A, Tomas D (2012). hGH and GHR expression in large cell neuroendocrine carcinoma of the colon and rectum. Anticancer Res.

[9] Minocha V, Shuja S, Ali R, Eid E (2014). Large cell neuroendocrine carcinoma of the rectum presenting with extensive metastatic disease. Case Rep Oncol Med.

[10] Xu F, Feng GS, Wang ZJ, Zhang KN (2014). Synchronous double cancers of colonic large cell neuroendocrine carcinoma and gastric squamous-cell carcinoma: a case report and review of literature. Int J Clin Exp Pathol.

[11] Kim JJ, Park SS, Lee TG, Lee HC, Lee SJ (2018). Large cell neuroendocrine carcinoma of the colon with carcinomatosis peritonei. Ann Coloproctol.

[12] Chetty R, Capo-Chichi JM, Serra S (2019). Colorectal large-cell neuroendocrine carcinoma with lymphoid stroma: further evidence confirming a unique subtype associated with MLH1/PMS2 loss, BRAF mutation, Epstein-Barr virus negativity, and the possibility of a better prognosis. Histopathology.

[13] La Rosa S, Marando A, Furlan D, Sahnane N, Capella C (2012). Colorectal poorly differentiated neuroendocrine carcinomas and mixed adenoneuroendocrine carcinomas: insights into the diagnostic immunophenotype, assessment of methylation profile, and search for prognostic markers. Am J Surg Pathol.

[14] Hashiguchi Y, Muro K, Saito Y, Ito Y, Ajioka Y, Hamaguchi T (2020). Japanese Society for Cancer of the Colon and Rectum (JSCCR) guidelines 2019 for the treatment of colorectal cancer. Int J Clin Oncol.

[15] Al-Talib A, Tulandi T (2010). Pathophysiology and possible iatrogenic cause of leiomyomatosis peritonealis disseminata. Gynecol Obstet Invest.

[16] Kumar S, Sharma JB, Verma D, Gupta P, Roy KK, Malhotra N (2008). Disseminated peritoneal leiomyomatosis: an unusual complication of laparoscopic myomectomy. Arch Gynecol Obstet.

[17] Akamine K, Kadono J, Otsuka H, Ueno K, Shimizu T, Nagata Y (2019). Gastrointestinal stromal tumor coexisting with disseminated peritoneal leiomyomatosis. Surg Case Rep.

[18] Moertel CG, Kvols LK, O'Connell MJ, Rubin J (1991). Treatment of neuroendocrine carcinomas with combined etoposide and cisplatin. Evidence of major therapeutic activity in the anaplastic variants of these neoplasms. Cancer.

[19] Mitry E, Baudin E, Ducreux M, Sabourin JC, Rufié P, Aparicio T (1999). Treatment of poorly differentiated neuroendocrine tumours with etoposide and cisplatin. Br J Cancer.

[20] Yamaguchi T, Machida N, Morizane C, Kasuga A, Takahashi H, Sudo K (2014). Multicenter retrospective analysis of systemic chemotherapy for advanced neuroendocrine carcinoma of the digestive system. Cancer Sci.

[21] Sorbye H, Welin S, Langer SW, Vestermark LW, Holt N, Osterlund P (2013). Predictive and prognostic factors for treatment and survival in 305 patients with advanced gastrointestinal neuroendocrine carcinoma (WHO G3): the NORDIC NEC study. Ann Oncol.

[22] Janson ET, Sorbye H, Welin S, Federspiel B, Grønbæk H, Hellman P (2014). Nordic guidelines 2014 for diagnosis and treatment of gastroenteropancreatic neuroendocrine neoplasms. Acta Oncol.

